# Study on Microbial Deposition and Contamination onto Six Surfaces Commonly Used in Chemical and Microbiological Laboratories

**DOI:** 10.3390/ijerph120708295

**Published:** 2015-07-17

**Authors:** Elena Tamburini, Valentina Donegà, Maria Gabriella Marchetti, Paola Pedrini, Cecilia Monticelli, Andrea Balbo

**Affiliations:** 1 Department of Life Science and Biotechnology, University of Ferrara, Ferrara, Via L. Borsari 46, 44121 Italy; E-Mails: dngvnt@unife.it (V.D.); mhm@unife.it (M.G.M.); pdp@unife.it (P.P.); 2 Corrosion and Metallurgy Study Centre “A. Daccò”, Department of Engineering, University of Ferrara, Via Saragat 4a, Ferrara 44122, Italy; E-Mails: cecilia.monticelli@unife.it (C.M.); andrea.balbo@unife.it (A.B.)

**Keywords:** surface contamination, *bioaerosol* deposition, surface roughness, total microbial counts

## Abstract

The worktops in both chemical and microbiological laboratories are the surfaces most vulnerable to damage and exposure to contamination by indoor pollutants. The rate at which particles are deposited on indoor surfaces is an important parameter to determine human exposure to airborne biological particles. In contrast to what has been established for inorganic pollutants, no limit has been set by law for microbial contamination in indoor air. To our knowledge, a comparative study on the effect of surfaces on the deposition of microbes has not been carried out. An evaluation of the microbial contamination of worktop materials could be of crucial importance, both for safety reasons and for the reliability of tests and experiments that need to be carried out in non-contaminated environments. The aim of this study was to evaluate the overall microbial contamination (fungi, mesophilic and psychrophilic bacteria, staphylococci) on six widely used worktop materials in laboratories (glass, stainless steel, fine porcelain stoneware, post-forming laminate, high-performing laminate and enamel steel) and to correlate it with the characteristics of the surfaces. After cleaning, the kinetics of microbial re-contamination were also evaluated for all surfaces.

## 1. Introduction

In modern urban settings, people spend more than 90% of their time in enclosed spaces, such as houses, office buildings and schools. Therefore, indoor contamination has caught the attention of scientists and the general public in many countries [1]. In most indoor environments, the air contains a variety of chemical and microbial particles, commonly defined as *indoor pollutants*, which can severely affect human health [2]. In particular, all particles and substances of biological origin or with biological activities diffused in the air are defined as *bioaerosol* [3], including bacteria, fungi, viruses, pollen and spores as well as their by-products (*i.e.*, toxins and allergens) [4]. Counting microbes in the air is not an easy task, and comparisons among different indoor environments are difficult due to the variability of the methods used in studies. There are still problems to be solved relating to the methodology, monitoring, data interpretation and maximum acceptable levels of contamination [5]. Concentration limits for the total number of *bioaerosol* particles in indoor locations are recommended by different agencies and organizations, *i.e.*, 1000 CFUs/m^3^, and the count for total living bacteria should not exceed 500 CFUs/m^3^ [6,7].

Measuring surface contamination could be more convenient than measuring air contamination, as sampling systems (*i.e.*, plates, swabs) are easily available and can be monitored without additional equipment [8]. The aim of this microbiological sampling is mainly to assess the contamination of a surface produced by the fallout of microorganisms from the air. The most reliable method to measure surface contamination is passive sampling since it provides a direct indication of the microbial contamination [9]. Several attempts to establish a relationship between culture counts in air and on surfaces have been carried out, and in some cases a linear regression was found [10].

Indoor deposition of *bioaerosol* is one of the most important factors that determines the adverse side effects of particle exposure on human health [11]. However, only a few studies have investigated microbial fallout on surfaces [12,13], and have been specifically directed towards understanding and quantifying the mechanisms controlling particle dynamics when an aerosol particle adheres to a surface upon contact [14]. 

When a microbe makes initial contact with a surface, the air turbulence and physical disturbance play a role in whether it will adhere or be re-aerosolized. In addition, the properties of the surface can influence the process, *i.e.*, moisture, hydrophilic interactions, electrostatic forces and physical configuration [15]. In the air, several microorganisms have difficulty maintaining viability, while deposition on a surface could assure the required humidity, temperature and nutrient availability for growth and sustainment of metabolic activities, which favors further contamination of surfaces [16]. Surface contamination is derived from airborne microorganisms, which may become re-aerosolized again and inhaled by workers or casual passers-by. They may be transferred by hand contact and contaminate food or be directly ingested [17]. 

This is particularly true in chemical and microbiological laboratories, where worktops are a critical element of the furniture, because they are the most vulnerable to mechanical damage, becoming more vulnerable to microbial deposition, and are largely exposed to contaminated indoor air and are usually located in highly frequented environments. 

Different types of worktop materials could be used, depending on specific needs, durability, maintainability and cost [18]. Today’s laboratories demand compliance with rigorous codes and standards [19], but guidelines are almost exclusively based on chemical resistance (*i.e.*, to corrosive substances). Microbial contamination of indoor air, unlike inorganic pollutants, still lacks acceptable exposure limits set by law. In work environments where the presence of potentially pathogenic agents can occur, an evaluation of the total microbial count (fungi and bacteria) is usually considered a sufficient index of contamination [20].

To our knowledge, a comparative study on the effect of surfaces on microbial deposition has not been carried out. However, an evaluation of the microbial contamination of worktop materials could be of crucial importance, both for safety reasons and the reliability of tests and experiments that need to be carried out in non-contaminated environments. Selecting the appropriate material for laboratory worktops and furniture is of crucial importance because tests and experiments often require non-contaminated environments. In fact, surface contamination can interfere with experiments solely by its existence or by increasing the background contamination levels.

The aim of this study was to evaluate and compare the overall microbial contamination (fungi, mesophilic and psychrophilic bacteria, staphylococci) on six widely used worktop materials in laboratories (glass, stainless steel, fine porcelain stoneware, post-forming laminate, high-performing laminate and enamel steel) exposed to identical environmental conditions in order to correlate the deposition of the bioaerosol with the characteristics of the different materials, e.g*.*, roughness. After cleaning with a detergent, the kinetics of microbial re-contamination were also evaluated for all surfaces.

## 2. Experimental Section 

### 2.1. Site and Worktop Surface Description

Samples of 40 × 40 cm of glass (G), stainless steel AISI 304 (INOX), fine porcelain stoneware (LG), post-forming laminate (PFL), high-performing laminate (HPL) and enamel steel (S) were placed close together in a chemical and microbiological laboratory of the University of Ferrara (Italy) for 30 days and used for normal activities. In the laboratory, chemical and GRAS (*Generally Recognized As Safe*) microbiological analyses were carried out daily by 3–4 occupants for 8 h per day. Four windows were opened daily for 1 h; thus, microbial contamination was derived from anthropic occupation and bioaerosols from outdoor air. The mean temperature over 30 days was 23 ± 3 °C and the relative humidity was 59.0 ± 7.0%, measured by an RS-1360° thermohygrometer (RS Components, UK).

### 2.2. Surface Sampling

After 30 days, surfaces were sampled using RODAC (*Replicate Organism Detection and Counting*) contact Petri plates (Liofilchem, Italy). Sedimented microorganisms were transferred directly to the plates via direct contact under standardized conditions (applying 0.02 kg/cm^2^ of constant pressure for 10 s). The plates had a surface area of 24 cm^2^ and a bottom grid to facilitate the counting of colonies. Mesophilic bacteria, psychrophilic bacteria, fungi and staphylococci were detected. Selected cultivation media were used for bacteria (Plate Count Agar at pH = 7.0 ± 0.2), fungi (Sabouraud Cloramfenicol Agar at pH = 5.6 ± 0.2), and staphylococci (Mannitol Salt Agar at pH = 7.4 ± 0.2) [21]. Mesophilic bacteria and staphylococci were incubated at 37°C, while the fungi and psychrophilic bacteria were incubated at 22 °C. 

All plates were incubated for 144 h, and the colony counts were registered at 24, 48, and 72 h. Microbial density was expressed in terms of CFUs (Colony Forming Units)/100 cm^2^ and calculated by dividing the count results (N, number of colonies per plate) by the contact area of the plate (24 cm^2^), and multiplying the result by 100. All sampling was carried out in triplicate.

### 2.3. Re-Contamination Kinetics

Surfaces were cleaned with a natural-based detergent (BluEsprit^®^, Eco12, Italy) using a paper towel, as is usually done in laboratories. The detergent is completely biodegradable, according to the OECD guidelines [22]. Samples were collected from surfaces every 24 h for six days. All plates were incubated for 144 h, and colony counts were registered at 24, 48, and 72 h. The surfaces were divided into 18 subareas such that 3 portions of the surface (separate from each other) were sampled per day. We assumed that contamination was equal across the entire surface.

After 144 h, an advanced stationary phase was consistently reached; therefore, we considered the values of the cell counts at that time the maximum values of the cell concentrations under these conditions and we used these for the kinetic calculations. 

The kinetics of microbial development were evaluated by calculating the maximum specific rate (μ) as follows: dx/dt = μt (where x is cell count and t is time) and doubling time (t_g_), calculated as t_g_ = ln2/μ. The doubling time, or generation time, is the period required for cells to double in quantity and it can be an indicator of the contamination rate of a surface.

**Figure 1:**
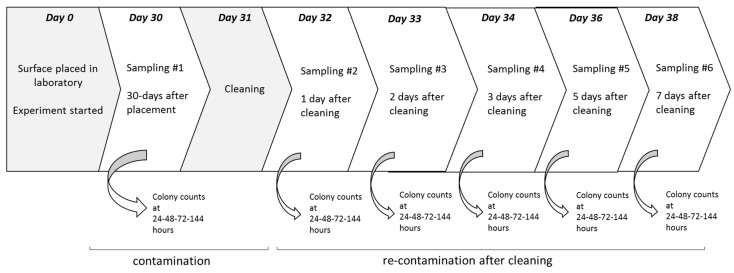
Schematic illustration of the experimental set up.

The overall experimental setting is presented in Figure 1.

### 2.4. Surface Roughness 

Surface topological characterization was carried out with a non-contact 3D profilometer (Taylor-Hobson CCI MP) over an area of 0.8 × 4 cm, at the center of each sample. The obtained topography data were analyzed using the commercial software Talymap 6.2.

The evaluation of 2D textural parameters (R_a_, R_t_) was performed on 4 different profiles extracted from the 3D data, and the gaussian filter (λ_c_) for the separation of the roughness and waviness component was set according to the ISO 4288:2000 [22]. The parameters were calculated as the averages of the estimated values for all sampling lengths over each profile.

## 3. Results and Discussion

### 3.1. Microbial Contamination of Surfaces

**Figure 2:**
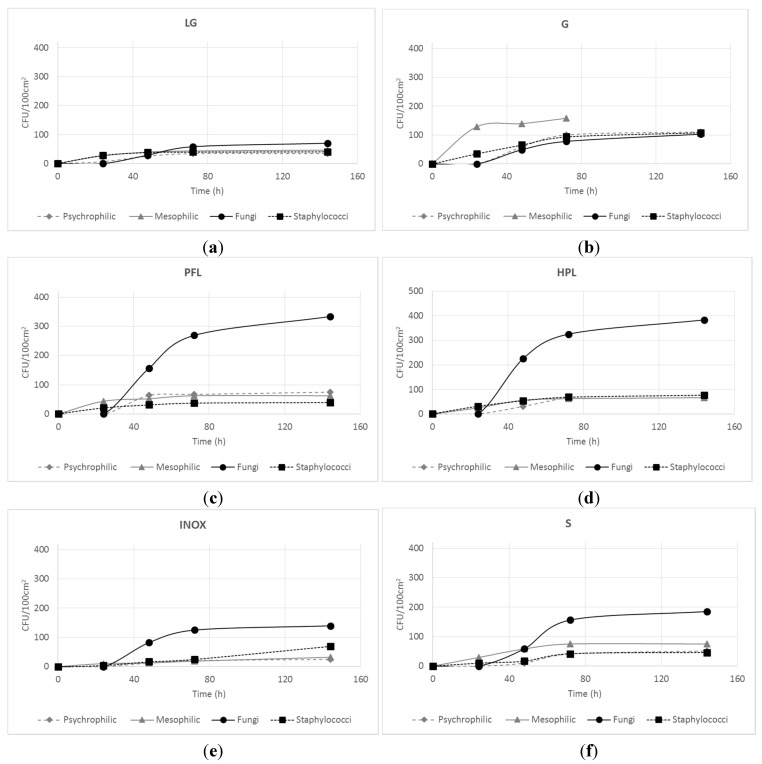
Increase in microbial counts after 30 days of environmental exposure for samples (**a**) fine porcelain stoneware (LG); (**b**) glass (G); (**c**) post-forming laminate (PFL); (**d**) high-performing laminate (HPL); (**e**) stainless steel (INOX); (**f**) enamel steel (S).

Sampling conducted after 30 days of identical environmental exposure, in terms of humidity, temperature and anthropic presence, revealed a slightly different microbial contamination level among the surfaces (Figure 2). All microorganisms showed a lag phase of about 24 h and similar growth trends, but different final cell concentrations. In particular, the fine porcelain stoneware exhibited the lowest contamination for all microbial groups (Figure 2A), whereas the post-forming laminate and high-performing laminate (Figure 2C,D) reached the highest microbial concentration after 144 h, principally due to fungal contamination. Except for glass, fungi were the dominant group of microorganisms on all surface types. This could be due to the fact that airborne fungal particles constitute the major component of ambient microorganisms, especially those that belong to the genera *Aspergillus* and *Penicillum* [23]. Because of their lower water requirements compared with bacteria, fungi are the principal contaminant on various types of substrates. They tend to colonize a wide variety of humid surfaces, wetted by floods or condensation [24]. Consequently, when fungal proliferation occurs, aerospores are abundantly distributed on and around the surfaces, and the indoor environment becomes a source of microbial exposure to its occupants. The deposition of fungal particles on surfaces is favored by the gravitational settling velocity that drives down the cells during their turbulent motion in air, increasing the probability of surface adhesion. Even though the temperature, humidity and substrate conditions of the surfaces would be rather hostile, the fungal spores remain in a quiescent state, ready to restart growing and re-contaminate the environment and humans when possible [25]. In fact, several studies have reported that indoor fungal concentration is significantly correlated with the occurrence of human diseases and public health problems, such as acute toxic effects, allergies, and asthma [26]. 

Mesophilic bacteria were the dominant group on glass (Figure 2B), both in terms of the final concentration and the viability. After incubation at 37 °C, these bacteria reached the exponential growing phase in a few hours and the stationary phase in only 24 h. 

**Figure 3:**
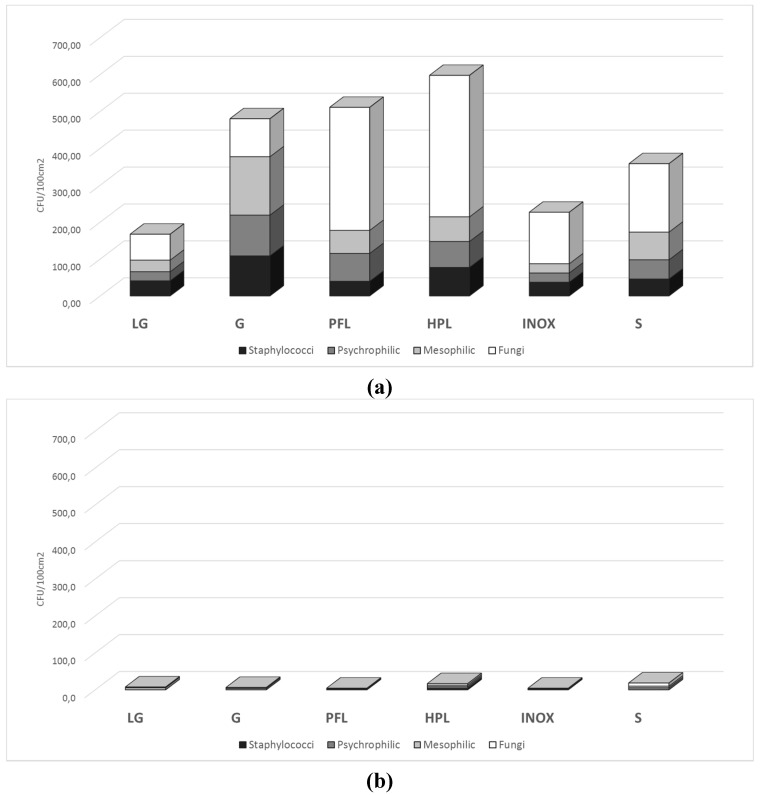
Total microbial counts (**a**) after 30 days of environmental exposure and (**b**) after the cleaning treatment.

After 30 days of normal laboratory practice, all microbial groups were present and viable on the six surfaces tested, although at different final cell concentrations. Figure 3A summarizes the final concentration (at 144 h) of the microbial groups monitored on the surfaces. Post-forming laminate, high-performing laminate and glass were the most contaminated, indicated by the TMC (Total Microbial Count), followed by enamel steel, stainless steel and fine porcelain stoneware. It is worth noting that fungi were the most represented (>60% of TMC), whereas the 3 bacterial groups (psychrophilic, mesophilic and staphylococci) were almost equally distributed in the remaining 40%. The presence of staphylococci, which accounted for 20% of TMC on all surfaces, confirms the anthropic presence in the laboratory. It is known that staphylococcal counts are useful indicators of human contamination, as they are part of the normal flora and can be found in the nose and other areas of the body. In locations frequented by humans, staphylococci can constitute up to 50% of the total mesophilic bacteria [27]. In all cases, staphylococci reached a concentration similar to that found in the air for mesophilic counts, confirming their distribution on the surfaces. 

In the glass sample, only 21% of TMC was represented by fungi, whereas the total bacterial counts constituted the remaining 79%, with 40% for the mesophilic group and 20% for staphylococci. 

The cleaning treatment with white paper towel and a detergent sprayed on the surfaces was able to reduce the TMC (Figure 3B) by more than 99%. The differences found for high-performing laminate and enamel steel were not statistically significant. Regardless of the initial level of contamination, the combination of mechanical and chemical effects of cleaning assured the temporary sanitization of surfaces by completely removing the microbial contamination amassed over 30 days. 

### 3.2. Microbial Re-Contamination of Surfaces after Cleaning Treatment

**Figure 4:**
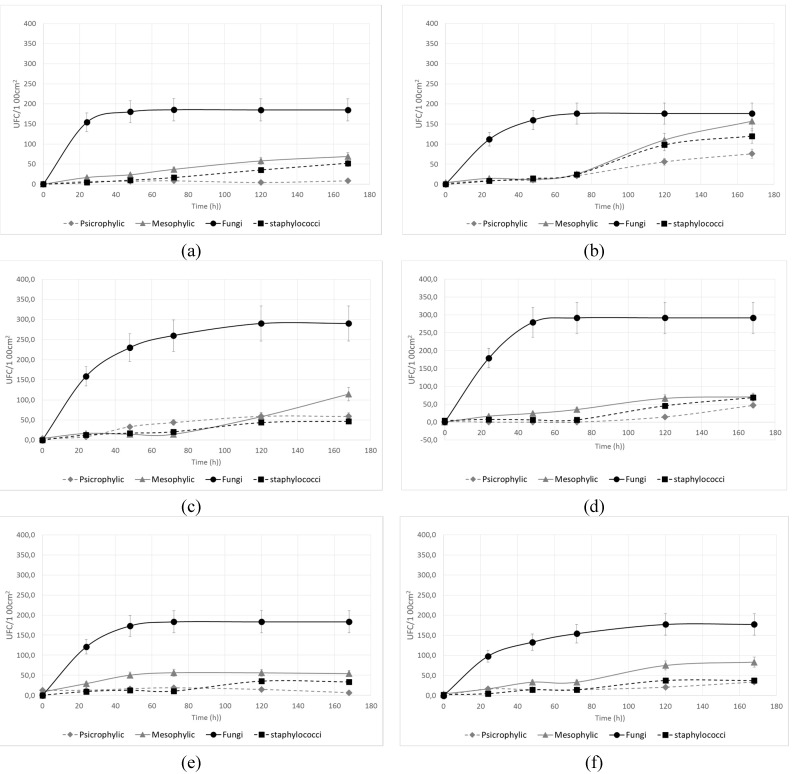
Microbial kinetics of re-contamination after the cleaning treatment for (**a**) fine porcelain stoneware (LG); (**b**) glass (G); (**c**) post-forming laminate (PFL); (**d**) high-performing laminate (HPL); (**e**) stainless steel (INOX); (**f**) enamel steel (S).

Each point on the graphs depicted in Figure 4, which corresponds to the final value of the cell counts obtained for the sampling surfaces after cleaning (*t* = 0) and for the subsequent 24, 48, 72, 120 and 168 h, was obtained by following the growth of the microorganisms for 144 h. As each sampling was an independent event, the standard deviations are reported. The trends represent the day-to-day increase in surface contamination, and it is worth noting that after 7 days, the overall cell counts already reached values comparable to the maximum detected after 30 days. 

Sampling using Petri dishes provides the microbial concentration in terms of overall viable cell counts, and we could not discriminate the contributions from previously deposited cells, quiescent cells and the new deposition of still viable cells. Moreover, in the case of permanent adhesion, the attached cells may start multiplying and form surface microcolonies, or they may remain quiescent even in the presence of organic material, particularly if there is little moisture available [28]. Regardless, the binding of microorganisms to a surface can confer advantages to cell survival, for example, the attachment of cells to solid surfaces has been reported to immediately upregulate alginate synthesis in a strain of *Pseudomonas* spp. [29], therefore strengthening cell-substratum binding.

Fungi reached a surface concentration plateau at most 72 h after cleaning, with a specific contamination rate of 0.191–0.216 h^−1^, corresponding to a doubling time of about 3 h (Table 1). Our results are consistent with the interval 0.08–1.08 h^−1^ found in the literature for *Aspergillus* spp. and *Penicillum* spp. deposition rates in the absence of ventilation [30]. For fungi, the deposition rate could be influenced by gravitational settling more than for bacteria, because they have a larger average aerodynamic diameter. Generally, the rate of deposition onto indoor surfaces is considerably smaller for particles with diameters in the range of 0.1–1 μm compared with particles with diameters in the range of 1–10 μm. Common indoor fungi and fungal spores have aerodynamic diameters of 2.2–7 μm, while bacteria are generally smaller (0.65–2 μm).

**Table 1:** Specific growth rate (μ) and generation time (t_g_) for the four microbial categories investigated.

Surface	Psychrophilic Bacteria		Mesophylic Bacteria		Fungi		Staphylococci	
	μ (h^−1^)	t_g_ (h)	μ (h^−1^)	t_g_ (h)	μ (h^−1^)	t_g_ (h)	μ (h^−1^)	t_g_ (h)
Fine porcelain stoneware (LG)	0.013	54.9	0.025	27.5	0.210	3.3	0.033	21.0
Post-forming laminate (PFL)	0.049	14.2	0.028	24.5	0.211	3.2	0.023	30.2
Stainless steel (INOX)	0.041	17.0	0.056	12.3	0.200	3.4	0.030	23.3
Glass (G)	0.056	12.4	0.065	10.6	0.197	3.5	0.064	10.8
High-performing laminate (HPL)	0.040	17.2	0.035	19.8	0.216	3.2	0.044	15.7
Enamel steel (S)	0.021	33.0	0.026	26.3	0.191	3.6	0.030	22.8

For example, it is reported that a 5–10-μm particle will fall 1 meter in 5–20 min in air, while a < 1-μm particle will fall 1 meter in 2–5 days [31]. Once deposited, fungal spores also have a significant probability to be re-suspended in air. Thatcher and Layton [32] have shown that re-suspension occurs predominantly for particles larger than 1 μm and that the amount of re-suspension increases with particle size. The plateau concentration of fungi could be explained by the rapid reaching of equilibrium between these two phenomena. 

Except for mesophilic bacteria on the INOX surface, all bacterial groups exhibited a “lag phase” of at least 72 h after cleaning before significant surface re-contamination. This could be due to the antibacterial activities of residues of the *Equisetum* extract-based detergent on the surface. Being completely biodegradable, its effect progressively vanished in 3–5 days, allowing redevelopment of bacteria. From 72 h onward, the specific rates of development of the 3 groups were similar, in the range of 0.013–0.056 h^−1^ for psychrophilic bacteria, 0.025–0.065 h^−1^ for mesophilic bacteria and 0.023–0.064 h^−1^ for staphylococci, corresponding to a doubling time of 12.4–54.9 h, 12.3–27.5 h and 10.8–23–3 h, respectively.

Among the surfaces, the glass appeared to be the most favorable for bacterial proliferation, with doubling times of surface contamination of approximately 10–12 h. Fine porcelain stoneware had the lowest specific rates of surface growth for mesophilic and psychrophilic bacteria, which also contributed to the lowest overall microbial counts after 7 and 30 days.

### 3.3. Effect of Surface Roughness on Microbial Deposition

Many studies have attributed microbial adhesion and survival on abiotic surface to the cellular physiology of microorganisms, but it is now known that several physical and chemical factors are involved in this process. The physicochemical parameters of the surface will affect initial adhesion. Once the cells attach, the surface chemistry influences cell adhesion, while topographic features affect the cell-surface binding, modifying the strength of the attachment and the subsequent probability of retention or re-suspension [33]. Even though microbial response to micro-scale features of surfaces is still controversial, several studies have showed that the most hygienic surfaces have low surface roughness values, while an increase in surface roughness favors the retention of microorganisms [34]. The different microbial behaviors observed in various studies could be attributed to the high variability of the species present in the bioaerosols and their relative concentrations; thus, it is difficult to compare the available data. Moreover, materials are often similar nominally (stainless steel, for example), but actually different in chemical composition and surface finishing, leading to significant variability in the experimental results obtained [35].

A high surface roughness protects against shear forces and increases convection transport, therefore facilitating microbial adhesion [36]. The adhesion could also be due to the enhancement of the cell-surface contact area, thereby allowing an increase in the binding energy [37]. Moreover, the shape of the cell, the rigidity of the membrane, the ability to form conglomerates or chains and to involve membrane structures allowing mobility and anchorage to the surface may affect microbial response to topographic features. In particular, flagella, pili, hyphae and other fimbriae may enhance the capability of microorganisms to create and maintain contact with the surface. Some authors have proposed that microbial response to nanometer scale roughness could be mediated by fimbriae-like structures and that flagella-related motility is of high importance for bacteria to reach the surface and to move into pores and/or recognize its topographical features [38]. 

Increasing the surface roughness facilitates the adhesion of microorganisms to the surface, especially when the average size of the surface pores is similar to the size of the microorganisms. Within surface pores, microorganisms find protection from environmental disturbances. Furthermore, rough surfaces favor the deposition of organic pollution, which in turn promotes the growth of microorganisms by providing nutrients. In general, surfaces with a surface roughness <0.8 μm are typically considered “hygienic” while roughness values >0.8 μm indicate higher susceptibility to the deposition of organic residues and microorganisms [39]. 

There are a number of engineering terms used to define surface roughness, but Ra (arithmetic mean value of the peak and valley distances measured along a centered line of the surface profile) and Rt (vertical distance from the deepest valley to the highest peak of the surface profile) are the most universally used roughness parameters in quality control [40] and microbiological publications [41].

**Figure 5:**
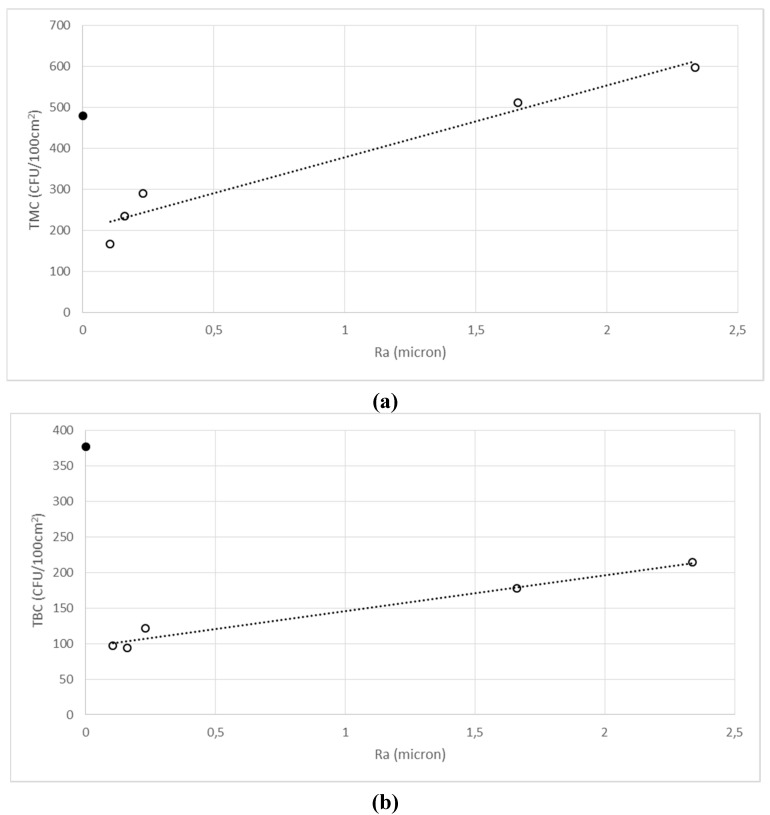

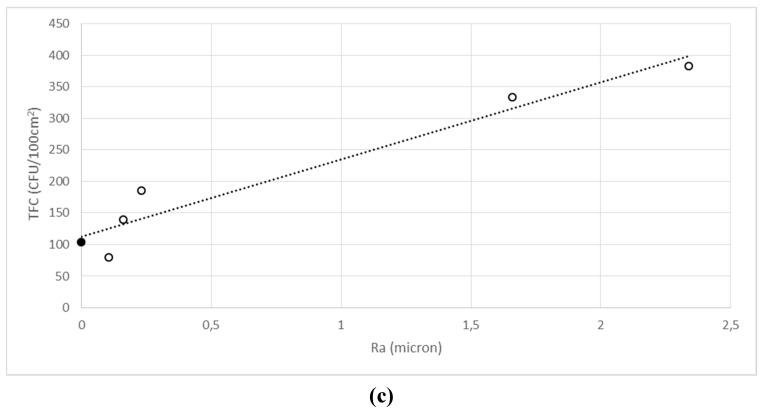
(**a**) TMC; (**b**) TBC and (**c**) TFC correlation *versus* surface roughness (Ra). The closed circle corresponds to the glass surface (G).

Both Ra and Rt values for the six surfaces are reported in Table 2, together with the total bacterial count (TBC), the total fungal count (TFC) and the total microbial count (TMC) calculated as the sum of TBC and TFC. As expected, positive correlations were found between the Ra and Rt values; therefore, for the sake of simplicity, only the microbial counts *versus* Ra relationships are reported in Figure 5.

TMC (Figure 5A) and TBC (Figure 5B) were linearly correlated (*R^2^* = 0.9598 and *R^2^* = 0.9661, respectively) with surface roughness for five surfaces (empty circles), but not the outlier glass (full circle), whilst TFC (Figure 5C) was linearly correlated (*R^2^* = 0.9451) with all six samples.

These results demonstrated that surface roughness can be considered the predominant surface characteristic influencing microbial adhesion, but in some cases, such as glass, other phenomena could play a role, especially for bacteria. Our results are in agreement with previously published data, which indicated a correlation between pore size and surface adhesion for values of surface roughness ranging from 0.03 to 8–9 μm [42]. The effect of glass on fungi is completely explainable by the dimensional interaction between surface pores and cell size [43]. Fungi are rather large cells that poorly adhere to smooth glass, reducing the probability of permanent adhesions. The unpredictable effect of glass on bacteria could be ascribed to surface wettability and electrostatic charge. Even though it has been reported that cell attachment to hydrophobic surfaces can occur rapidly [44], hydrophilic surfaces, such as glass, display better affinity for cells, particularly bacteria. It has been shown that surface hydrophilicity favors the adhesion of vegetative cells [45], due to hydrogen bond formation and Van der Waals interactions between the cells and the surface. Furthermore, an increased affinity for water could transform the surfaces into a more suitable environment for microbial growth [46]. Glass without any surface treatments is highly hydrophilic and is characterized by high wettability due to the tendency to form hydrogen bonds with the water molecules present in the environment [47]. Moreover, the untreated glass has a net negative surface charge and thus, according to the DLVO (Derjaguin, Landau, Verwey e Overbeek) theory [48], attracts bacteria. The DLVO theory describes the net cell-surface interaction as a balance between attractive and repulsive forces acting at the interface of the substratum and the cell membrane, which is positive due to the negative charge inside [49].

Stainless steel is a corrosion-resistant alloy (CRA) due to a nanometric surface film of oxides/hydroxides of chromium and iron (passive film) that protects and drastically reduces their corrosion rate. The nature of the passive film is quite complex and depends on the type of environment to which the alloy is exposed [50]. It is well known that the surface film, when exposed to an atmospheric environment or aqueous solution, interacts with water molecules and an outermost layer of hydroxyl groups is formed [51]. The surface charge of a passive alloy depends on many factors such as the nature of the passive film, the acid-base equilibria that are established in the outermost layer, the pH, the presence of ions specifically adsorbed and environmental pollutants. The isoelectric point (IEP) can provide a good indication of the state of the surface: if the pH is less than the IEP, the surface will acquire a positive charge, if the pH is greater than the IEP, the surface is negatively charged; for pH values close to the isoelectric point, the zeta potential is small, as well as the surface charge, and consequently, the electrostatic phenomena play a secondary role in the bioadhesion. 

The literature data indicate IEP values for AISI 304 varying between 3.8 and 5.5, depending on the environment and the surface finishing [52,53,54]; therefore, in a natural environment near neutrality, stainless steel exhibits a relatively low negative charge (zeta potential values around 10–20 mV). The surface of the INOX samples, exposed to laboratory atmosphere at moderately low values of relative humidity (59%), is covered by a nearly monolayer of water molecules and a low surface charge is expected. Under these conditions, the effect of surface charge in the bioadhesion process is limited.

High-performing laminate and post-forming laminate have a neutral charge; therefore, the electrostatic forces did not have an effect on microbial adhesion, which was driven almost exclusively by roughness. 

**Table 2:** Microbial contamination expressed as Total Bacterial Count (TBC); Total Fungal Count (TFC), Total Microbial Count (TMC) and roughness characterization of the surfaces as Ra and Rt values.

Surface	TBC (UFC/100 cm^2^)	TFC (UFC/100 cm^2^)	TMC (UFC/100 cm^2^)	Ra (Micron)	Rt (Micron)
Fine porcelain stoneware (LG)	96.89 ± 15.23	70.30 ± 20.12	167.19 ± 32.96	0.102 ± 0.004	1.813 ± 0.186
Post-forming laminate (PFL)	177.43 ± 25.56	333.35 ± 45.58	510.82 ± 47.00	1.660 ± 0.108	3.030 ± 0.735
Stainless steel (INOX)	94.20 ± 18.52	139.58 ± 33.69	233.78 ± 45.56	0.160 ± 0.008	13.556 ± 1.850
Glass (G)	377.05 ± 45.78	103.02 ± 17.56	480.05 ± 57.55	0.00020 ± 1.09E-05	0.019 ± 0.006
High-performing laminate (HPL)	214.15 ± 35.41	383.33 ± 54.50	597.48 ± 77.68	2.337 ± 0.074	20.075 ± 2.605
Enamel steel (S)	122.00 ± 19.12	185.41 ± 34.89	303.87 ± 38.22	0.197 ± 0.051	4.220 ± 1.225

## 4. Conclusions 

Traditionally, the efforts to control air pollution focused on outdoor air, but it is now apparent that contaminants are common inside buildings and are present on surfaces. Understanding how indoor pollution can affect indoor environments implies being familiar with the methodologies for monitoring the indoor air and the surface quality, and interpreting the results in the context of operators’ exposure. 

This study has shown that surface characteristics greatly influence surface susceptibility to microbial deposition. When exposed to the same environmental conditions of temperature, relative humidity, anthropic presence and air quality, the six surfaces examined had different effects, which were highly dependent on their different surface roughnesses. In the case of glass, other aspects, such as electrostatic forces and higher wettability, resulted in an unexpected attraction for bacteria. The best roughness-contamination ratio was obtained for the fine porcelain stoneware and stainless steel, which consequently could be considered the best solutions to decrease surface microbial contamination occurrence in microbial laboratories. In other laboratories, all surfaces could be used, taking into account that at least every week a deep cleaning should be carried out, because in 7 days, microbial concentration on surfaces reaches a maximum level, increasing the probability of re-suspension phenomena or surface over-contamination, which could have effects on human health and operators’ safety. Microbial particles can cause breathing problems and allergies in sensitive individuals and potential severe health problems for persons with asthma. Controlling the concentration of particulates through cleaning can help relieve symptoms and prevent disease. Health is a state of complete physical, mental and social well-being, not merely the absence of disease or infirmity [55]. Maintaining human health depends on a balanced interaction between many environmental factors, among which the quality of indoor air and that of surfaces are fundamental aspects. 
